# Supplementary material for: Cross-Cultural Adaptation and Validation of the Oral Health Values Scale for the Portuguese Population
Source: J Pers Med. 2022 Apr 22;12(5):672. doi: 10.3390/jpm12050672 (PMC9143491; doi:10.3390/jpm12050672)
Supplement: Supplementary file 1 [file jpm-12-00672-s001.zip › jpm-1684665-supplementary.pdf]

Journal of *Personalized Medicine*

Article

Cross-Cultural Adaptation and Validation of the Oral Health Values Scale for the Portuguese Population

Online Supplemental Information

Vanessa Machado [1,2,*,†], André Mendonça [1,†], Luís Proença [2,3], José João Mendes [1,2], João Botelho [1,2], Daniel W. McNeill [4] and Ana Sintra Delgado [1,5]

[1] Clinical Research Unit (CRU), Centro de Investigação Interdisciplinar Egas Moniz (CiiEM), Egas Moniz—Cooperativa de Ensino Superior, 2829-511 Monte da Caparica, Portugal; andremendonca@outlook.pt (A.M.); jmendes@egasmoniz.edu.pt (J.J.M.); jbotelho@egasmoniz.edu.pt (J.B.); anasintradelgado@gmail.com (A.S.D.)
[2] Evidence-Based Hub, CiiEM, Egas Moniz—Cooperativa de Ensino Superior, 2829-511 Monte da Caparica, Portugal; lproenca@egasmoniz.edu.pt
[3] Quantitative Methods for Health Research (MQIS), CiiEM, Egas Moniz—Cooperativa de Ensino Superior, 2829-511 Monte da Caparica, Portugal
[4] West Virginia University, Morgantown, WV 26506, USA; dmcneil@wvu.edu
[5] Orthodontic Department, Egas Moniz Dental Clinic (EMDC), Egas Moniz—Cooperativa de Ensino Superior, 2829-511 Monte da Caparica, Portugal
* Correspondence: vmachado@egasmoniz.edu.pt
† These authors contributed equally to this work.

Table S1. Test–retest reliability using Cronbach α coefficient and intraclass correlation coefficient for each item of OHVS-PT questionnaire.

	Cronbach's α coefficient (95% CI)	ICC (95% CI)	p-value
Item 1	0.95 (0.83; 1.00)	0.91 (0.82; 0.96)	<0.001
Item 2	0.91 (0.79; 0.98)	0.84 (0.68; 0.922)	<0.001
Item 3	0.87 (0.55; 1.00)	0.77 (0.56; 0.88)	<0.001
Item 4	0.95 (0.56; 1.00)	0.90 (0.80; 0.95)	<0.001
Item 5	0.94 (0.83; 0.99)	0.88 (0.76; 0.94)	<0.001
Item 6	0.83 (-0.09; 0.99)	0.72 (0.48; 0.86)	<0.001
Item 7	0.84 (0.46; 0.96)	0.73 (0.49; 0.86)	<0.001
Item 8	0.91 (0.76; 0.98)	0.84 (0.68; 0.92)	<0.001
Item 9	0.97 (0.93; 0.99)	0.94 (0.88; 0.97)	<0.001
Item 10	0.96 (0.88; 0.99)	0.92 (0.83; 0.96)	<0.001
Item 11	0.84 (0.43; 1.00)	0.72 (0.48; 0.86)	<0.001
Item 12	0.84 (0.58; 1.00)	0.73 (0.50; 0.87)	<0.001

Abbreviations: CI - confidence interval; ICC - intraclass correlation coefficient.
